# The association of low serum uric acid with mortality in older people is modified by kidney function: National Health and Nutrition Examination Survey (NHANES) 1999–2018

**DOI:** 10.1186/s12882-024-03546-6

**Published:** 2024-03-19

**Authors:** Zhongcheng Fan, Zhongju Li, Aixin Guo, Yang Li

**Affiliations:** 1 Department of Osteology, Haikou Municipal People’s Hospital and Central South University Xiangya Medical College Affiliated Hospital, Haikou, China; 2 Division of Ultrasonography, Haikou Municipal People’s Hospital and Central South University Xiangya Medical College Affiliated Hospital, Haikou, China; 3 DHC Technologies, Beijing, China; 4 Division of Nephrology and Rheumatology, Haikou Municipal People’s Hospital and Central South University Xiangya Medical College Affiliated Hospital, 43 Renmin Ave, Haikou, 570208 China

**Keywords:** Uric acid, Older people, Mortality, Cardiovascular, Kidney function

## Abstract

**Background:**

In older individuals, the role of low serum uric acid (SUA) as risk factor for mortality is debated. We therefore studied whether SUA levels, particularly low SUA concentrations, are associated with all-cause and cardiovascular (CV) mortality in older population, and to clarify potential effect modification of kidney function.

**Methods:**

We identified 14,005 older people in National Health and Nutrition Examination Survey (NHANES) data from 1999 to 2018. SUA was measured only at baseline. The relationship between SUA and mortality was assessed using Cox proportional hazards models and restricted cubic spline Cox regression stratified by the estimated glomerular filtration rate (eGFR).

**Results:**

During mean 8.3 years of follow-up, 4852 all-cause death and 1602 CV death were recorded. A significant U-shaped association was observed between SUA with all-cause mortality, with the lowest risk concentration of 5.5 mg/dL. Comparing to the reference group (5 to 7 mg/dL), the HR of 2 to < 5 mg/dL group was 1.11 (1.03–1.21) and 1.14 (1.00–1.30). This relationship was more pronounced in participants with an eGFR ≥ 60 ml/min/1.73m^2^ (HR, 1.16; 95%CI, 1.06—1.28). This situation similarly occurred in Urine protein negative group (HR, 1.14; 95%CI, 1.04—1.25).

**Conclusions:**

Low SUA concentrations are associated with an increased risk in all-cause and CV mortality among older participants. Extremely low SUA concentrations are especially undesirable, especially in the older adults with normal kidney function.

## Introduction

With population ageing, the health problem of the older people is more prominent. Cardiovascular (CV) disease is the leading cause of death in older people and increases exponentially with age. Exploring modifiable risk factors can help promote health and longevity in older adults.

Serum uric acid (SUA) has gained increasing attention as a CV risk factor [1]. A wealth of researches have focused on an elevated SUA level with higher risk of death from any causes and CV disease in adults [2, 3], while the relationship of low SUA in older people are inconclusive [4, 5]. Recently, there is some evidence suggesting a link between low uric acid levels and poor survival outcomes in elderly individuals [6], more research is needed to fully evaluate this relationship.

The prevalence of Chronic kidney disease (CKD) in older population increased, and this condition often affects SUA levels, due to the insufficient excretion of uric acid [7, 8]. There are some evidence that the presence of CKD attenuates the strength of association between SUA and mortality compared to that observed among persons without CKD [9].

In older adults, concomitant conditions such as multimorbidity, frailty, disability and appetite regulation may confound the association between CV risk factors and adverse outcomes, leading to risk factor reversal [10]. Thus, the SUA intervention threshold identified in younger individuals may not apply to these older ones. We therefore studied whether SUA levels, particularly low SUA concentrations, are associated with all-cause and CV mortality in older population, and to clarify potential effect modification of reduced kidney function.

## Methods

### Study population

In order to monitor the health and nutritional status of the US civilian non-institutionalized population, the National Health and Nutrition Examination Survey (NHANES) as a largescale, multistage and ongoing was performed by the National Center for Health Statistics of the Centers for Disease Control and Prevention (CDC). To produce reliable statistics, NHANES over-samples persons 60 and older, and the older the individual, the more extensive the examination [11].

We used of ten cycles of NHANES data from 1999 to 2018. There are 19,056 individuals aged 60 years or older for whom follow-up data were eligible. We excluded participants who present of gout (*n* = 1136), dialysis in the previous 12 months (*n* = 96), with missing information and abnormal value on age, sex, race, education, BMI, biochemical test, comorbidities, co-medications (*n* = 3748), died or lose follow-up within 6 months (*n* = 71). Therefore, a total of 14,005 participants entered in our present analysis.

### Outcomes

Study outcomes included any cause (all-cause) mortality and CV mortality. Every participant was follow up to 31 December 2019 or death, which one came first.

The causes of death was ascertained by matching to the National Death Index (NDI), a database of all deaths in the United States. CV mortality was defined as the primary cause of death being any disease of the circulatory system (ICD-10 codes I00-I09, I11, I13, I20-I51, or I60–I69).

### Assessment of variates

Information on age, sex, race/ethnicity, education levels, smoking status, comorbidities, and medication use was collected from household interviews using standardized questionnaires. Body weight and height were obtained when people participated in the physical examinations at a mobile examination center. BMI was calculated as weight in kilograms divided by height in meters squared. Race/ethnicity was classified as non-Hispanic White, non-Hispanic Black, Mexican American, or other. Education level was categorized as low than high school, high school or equivalent, or college or above. Serum uric acid, creatinine, triglycerides, total cholesterol, high density lipoprotein cholesterol, urine albumin and creatinine concentrations were measured at baseline when the participants provided their blood and urine samples. Urinary albumin and creatinine concentrations were measured by solid phase fluorescence immunoassay and Jaffe rate reaction, respectively, in random single urine samples. urinary albumin-creatinine ratio (UACR) was calculated by urinary albumin divided by urinary creatinine. eGFR value was calculated using the creatinine equation developed by the Chronic Kidney Disease Epidemiology Collaboration (CKD-EPI) [12].

Diabetes was defined as self-reported doctor diagnosis of diabetes, use of insulin or oral hypoglycemic medication, fasting glucose ≥ 7.0 mmol/L, random glucose ≥ 11.1 mmol/L, or glycated hemoglobin A1c (HbA1c) ≥ 6.5%. Hypertension status was obtained from self-report, or systolic blood pressure ≥ 140 mm Hg or diastolic blood pressure ≥ 90 mm Hg. ASCVD risk was calculated according ACC/AHA Guideline [3]. CCI was calculated based on the literature published in 1997 [4].

### Statistical analyses

Sample weights, clustering, and stratification were incorporated in all analyses because of the complex sampling design of the NHANES, as required to analyze the NHANES data [11]. Participants was followed to the date 31 December 2019 or death, whichever came first.

The study participants were categorized into three groups as 2 to < 5, 5 to < 7, and ≥ 7 mg/dL. Baseline characteristics are summarized as means (standard error) for continuous variables and numbers (percentages) for categorical variables.

Relationships between SUA concentrations and the risk of outcomes were evaluated on a continuous scale with restricted cubic spline (RCS) curves with 3 knots (the smallest Akaike information criterion) based on Cox proportional hazards regression models with adjustment for age, sex, race, education, smoke status, BMI, WBC count, hemoglobin, serum albumin, total cholesterol, triglycerides, urine albumin to creatinine ratio, eGFR, ASCVD risk, comorbidities (hypertension, diabetes, CV disease, CCI), and co-medications (anti-hypertension, lipid-lowering drugs, hypoglycemic, urate-lowing agents).

Associations of SUA level with all-cause and CV mortality were investigated using Cox proportional hazards regression models, with adjustment for confounders, age, sex, race, education, smoke status, BMI, laboratory test results, ASCVD risk, comorbidities, and co-medications. Hazard ratios (HR) and 95% confidence intervals (CI) were estimated for each SUA category.

We further performed subgroup analysis stratified by baseline characteristics including eGFR (< 60 and ≥ 60 ml/min/1.73 m^2^), UACR(< 30 and ≥ 30 mg/g), with or without CV disease and obesity (BMI ≥ 30 kg/m^2^).

Additional sensitivity analysis was conducted to verify the robustness of the results: considering the different survey years may affect the treatment strategies, we performed another analysis with adjusting survey years (survey cycles).

All analyses were carried out with R (package survey, version 4.2.0), a two-sided *p* value of less than 0.05 was considered as statistically significance.

## Results

After applying all exclusion criteria, a total of 14,005 eligible participants were included in the present study, representing 44 million old people in the United States. The baseline characteristics of the study cohort stratified by the SUA concentration are presented in Table 1. The population had a mean age of 69.8 years, included 7181 (51.3%) females. The SUA concentrations were mainly found to be between 5 and 7 mg/dL (49.9%) in the data set. A total of 69.8%, 30%, and 22.7% of the participants had hypertension, diabetes mellitus, and CV disease, respectively. Compared with the lowest SUA level (2 to < 5 mg/dL), patients with higher SUA levels were more likely to be male, black, former or current smoker, obesity (BMI ≥ 30 kg/m^2^), had increased WBC count, UACR, triglycerides, and prevalence of comorbidities (hypertension, diabetes, CV disease and CCI count); however, patients with higher SUA concentrations had a lower eGFR and a reduced total cholesterol level, for all these comparisons (*P* < 0.001).

**Table 1:** Baseline characteristics stratified by SUA level

Characteristics^a^*n*	All	SUA, mg/dL^b^		
		**2 to < 5** **(*n* = 4607)**	**5 to < 7** **(*n* = 6990)**	** ≥ 7** **(*n* = 2408)**
Age, yr	69.8 (0.1)	69.5 (0.1)	69.8 (0.1)	70.7 (0.2)
Female, n (%)	7181 (51.3)	3200 (74.9)	3153 (48.9)	828 (37.5)
Race, n (%)				
White	7278 (52)	2354 (79.9)	3690 (80.3)	1234 (77.8)
Black	2537 (18.1)	628 ( 5.9)	1268 ( 7.7)	641 (11.6)
Mexican American	2203 (15.7)	895 (4.9)	1046 (3.7)	262 (3.0)
Other	1987 (14.2)	730 (9.3)	986 (8.3)	271 (7.6)
Education, n (%)				
Low than high school	4750 (33.9)	1608 (20.8)	2316 (21.0)	826 (24.6)
High school	3315 (23.7)	1061 (25.9)	1679 (26.4)	575 (25.4)
College	5940 (42.4)	1938 (53.4)	2995 (52.5)	1007 (50.0)
Smoke, n (%)				
Never	6869 (49.1)	2553 (54.3)	3331 (48.4)	985 (40.4)
Former	5343 (38.2)	1441 (33)	2749 (39.7)	1153 (50.3)
Current	1793 (13)	613 (12.8)	910 (11.9)	270 ( 9.4)
BMI, kg/m^2^				
< 25	3747 (26.8)	1705 (39.8)	1665 (22.1)	377 (14.9)
25 to < 30	5318 (38)	1739 (36.2)	2688 (38.8)	891 (35.8)
≥ 30	4940 (35.3)	1163 (24)	2637 (39.0)	1140 (49.3)
WBC, cells × 10^9^/L	7.07 (0.0)	6.86 (0.1)	7.09 (0.1)	7.46 (0.1)
Hemoglobin, g/dL	14.10 (0.0)	13.87 (0.0)	14.24 (0.0)	14.17 (0.1)
Serum albumin, g/L	41.99 (0.1)	41.96 (0.1)	42.08 (0.1)	41.79 (0.1)
TCHO, mmol/L	5.15 (0.0)	5.28 (0.0)	5.12 (0.0)	4.98 (0.0)
TG, mmol/L	1.73 (0.0)	1.55 (0.0)	1.76 (0.0)	1.99 (0.0)
UACR, mg/g	48.68 (2.8)	28.96 ( 2.3)	42.42 ( 2.9)	110.51 (13.2)
eGFR, ml/min/1.73m^2^	73.68 (0.2)	80.13 (0.3)	72.94 (0.3)	62.25 (0.5)
ASCVD risk	0.28 (0.0)	0.30 (0.0)	0.26 (0.0)	0.27 (0.0)
CCI, count	1.65 (0.0)	1.56 (0.0)	1.61 (0.0)	1.98 (0.1)
Hypertension, n (%)	9770 (69.8)	2912 (58.9)	4880 (67.4)	1978 (81.3)
Diabetes, n (%)	4198 (30)	1263 (21.4)	2075 (24.8)	860 (32.5)
CV disease, n (%)	3176 (22.7)	819 (17.0)	1579 (21.8)	778 (32.3)
Anti-hypertension, n (%)	7164 (51.2)	1893 (40.3)	3560 (50.1)	1711 (70.7)
Lipid-lowering drugs, n (%)	5274 (37.7)	1594 (36)	2684 (41)	996 (44.0)
Hypoglycemic, n (%)	2741 (19.6)	846 (14.0)	1335 (15.8)	560 (21.9)
Urate-lowing agents, n (%)	170 (1.2)	50 (1.2)	85 (1.1)	35 (1.3)

*Abbreviations*: *SUA* serum uric acid, *BMI* body mass index, *TCHO* total cholesterol, *TG* triglycerides, *UACR* urine albumin to creatinine ratio, *eGFR* estimated glomerular filtration rate, *CKD* chronic kidney disease, *CV* cardiovascular, *ASCVD* atherosclerotic cardiovascular disease, *CCI* Charlson Comorbidity Index

^a^All estimates accounted for complex survey designs. Continuous variables were expressed as mean (standard error). Categorical variables were expressed as number (percent)

^b^All intervals are left closed and right open

**Fig. 1:**
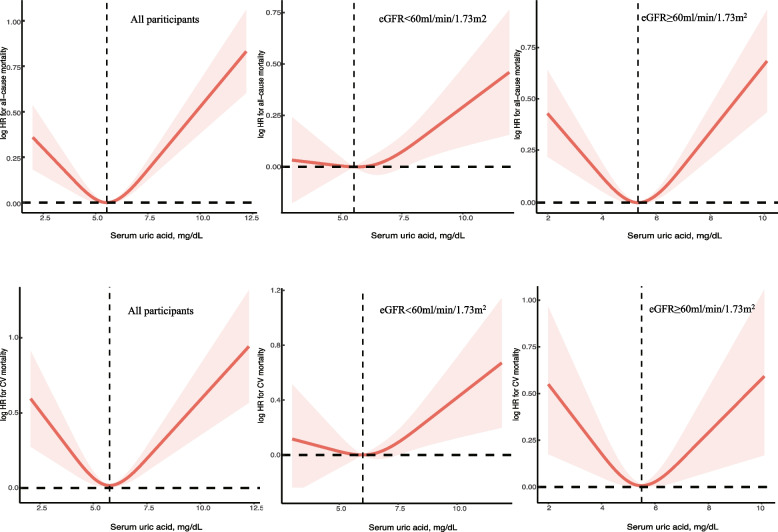
Relation of SUA with all-cause and CV mortality stratified by eGFR. Hazard ratio (HR) was adjusted for age, sex, race, education, smoke status, BMI, WBC count, hemoglobin, serum albumin, total cholesterol, triglycerides, urine albumin to creatinine ratio, eGFR, ASCVD risk, comorbidities (hypertension, diabetes, CV disease, CCI), and co-medications (anti-hypertension, lipid-lowering drugs, hypoglycemic, urate-lowing agents). Abbreviation: CV, Cardiovascular

During mean 8.3 years of follow-up, 4852 all-cause death (including 1602 CV death) were observed in this cohort, which included 1458 (28.2%) patients in the SUA 2 to < 5 mg/dL group, 2307(28.6%) patients in the SUA 5 to < 7 mg/dL group, and 1087(41.9%) patients in the SUA ≥ 7 mg/dL group, respectively. After multivariable adjustment, a significant U-shaped association was observed between SUA with all-cause and CV mortality, with the lowest risk concentration of 5.5 mg/dL and 5.6 mg/dL (Fig. 1). This relationship still obvious in participants with eGFR ≥ 60 ml/min/1.73m^2^, but not with eGFR < 60 ml/min/1.73m^2^.

Comparing with the reference group (5 to < 7 mg/dL), there was a higher risk of both all-cause and CV mortality when the SUA exceeded 7 mg/dL, the HR was 1.25 (1.13—1.38) and 1.25 (1.08—1.44). Notably, the risk of all-cause and CV morality also increased when the SUA was lower than 5 mg/dL, the HR was 1.11 (1.03—1.21) and 1.14 (1.00—1.30), further confirming the non-linear relationship between SUA concentration and mortality risk (Table 2).

**Table 2:** The association between SUA with all-cause and CV mortality

**SUA, mg/dL**	**Follow-up, years**	**All-cause mortality**			**CV mortality**		
		**Events** **(incidence rate)**	**Adjusted HR** **(95% CI)**^**a**^	***P* value**	**Events** **(incidence rate)**	**Adjusted HR** **(95% CI)**^**a**^	***P* value**
2 to < 5	8.4 (0.1)	1458 (28.2)	1.11 (1.03, 1.21)	0.01	445 (8.3)	1.14 (1.00, 1.30)	0.05
5 to < 7	8.3( 0.1)	2307 (28.6)	Reference	-	763 (9.4)	Reference	-
≥ 7	7.9 (0.2)	1087 (41.9)	1.25 (1.13, 1.38)	< 0.001	394 (15.1)	1.25 (1.08, 1.44)	0.003

*Abbreviations*: *CV* cardiovascular, *SUA* serum uric acid, *HR* hazard ratio, *CI* confidence interval, *ASCVD* atherosclerotic cardiovascular disease, *CCI* Charlson Comorbidity Index

^a^Adjusted for age, sex, race, education, smoke status, BMI, WBC count, hemoglobin, serum albumin, total cholesterol, triglycerides, urine albumin to creatinine ratio, eGFR, ASCVD risk, comorbidities (hypertension, diabetes, CV disease, CCI), and co-medications (anti-hypertension, lipid-lowering drugs, hypoglycemic, urate-lowing agents)

Effect modification by eGFR (< 60 and ≥ 60 ml/min/1.73m^2^) was observed (p for interaction 0.048). Among participants with normal kidney function, the association of SUA and all-cause mortality were remained U-shaped: compared with levels of 5 to 7 mg/dL, HR was 1.16 (1.06–1.28) in the 2 to 5 mg/dL group and 1.30(1.13–1.50) in the ≥ 7 mg/dL group, respectively. But in elders with CKD, this U-shaped association changed, the 2 to < 5 group was no longer associated with an increased risk of death (HR 0.94, 95%CI 0.80—1.10). This situation similarly occurred in UACR subgroup (< 30 and ≥ 30 mg/g), while UACR more than 30 mg/g, the association of 2 to < 5 level and mortality was attenuated to 0.99 (0.85–1.16).

**Fig. 2:**
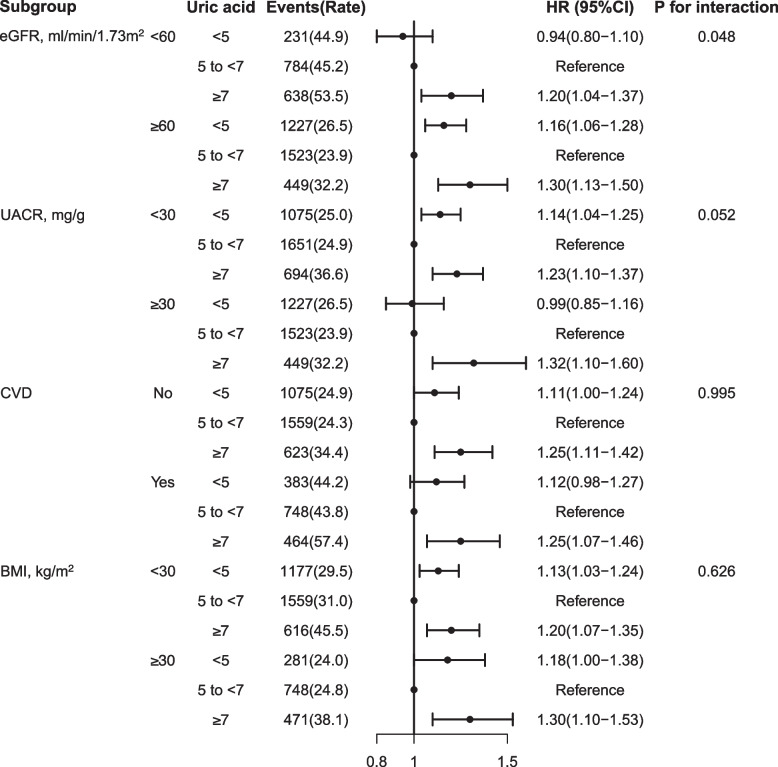
The association between SUA levels and all-cause mortality in various subgroups. Hazard ratio (HR) was adjusted for age, sex, race, education, smoke status, BMI, WBC count, hemoglobin, serum albumin, total cholesterol, triglycerides, urine albumin to creatinine ratio, eGFR, ASCVD risk, comorbidities (hypertension, diabetes, CV disease, CCI), and co-medications (anti-hypertension, lipid-lowering drugs, hypoglycemic, urate-lowing agents). Abbreviations: eGFR, estimated glomerular filtration rate; CV, Cardiovascular; UACR, urine albumin to creatinine ratio

The U-shaped association between SUA concentration and all-cause mortality was consistent with or without CV disease and obesity (Fig. 2).

We have performed another analysis with adjusting survey years (survey cycles). The results still supported the conclusions of this study. After multivariable adjustment, a significant U-shaped association was observed between SUA with all-cause and CV mortality.

## Discussion

This study showed a significant U-shaped association between SUA concentration with all-cause and CV mortality, with the lowest mortality rate occurring in individuals with SUA concentrations ranging from 5 to 7 mg/dL (included 49.9% individuals), while revealing considerable eGFR-related differences (< 60 and ≥ 60 ml/min/1.73m^2^): the positive association of high uric acid levels (≥ 7 mg/dL) with all-cause mortality were strong and consistent, however the relationship of low uric acid levels (2 to < 5 mg/dL) with mortality was disappeared in elders combined with eGFR < 60 ml/min/1.73m^2^.

Previous studies on the association of SUA with mortality in the older people are limited and inconclusive. Some studies have suggested that SUA at the top quantile or > 7.0 mg/dL is associated with a higher risk for CV mortality in older adults [4, 13]. However, these data failed to find an increased risk of death in older adults at low concentrations of SUA [4, 13]. In some studies of older adults, SUA has not even been proven as a risk factor for cardiovascular disease [14]. Only few investigations came to know a slight increased risk of mortality in older patients with low SUA level, among those special individuals, such as diabetes [15] and malnutrition [6].

Our study found that an SUA level 2 to < 5 mg/dL was associated with greater mortality risks in the elderly population. This finding is particularly important because current guidelines do not provide an optimal lower limit for SUA concentrations [16, 17]. The URRAH study [18] even confirm that SUA levels are linearly associated with an increased risk of all-cause and CV mortality, independently from common cardiovascular risk factors, with the threshold for increasing the risk of total and CV mortality are 4.7 mg/dL and 5.6 mg/dL, significantly lower than those used for the definition of hyperuricemia in current clinical practice, this may not apply to elders. Low SUA concentration could reflect weakness and malnutrition, so the present analysis excluded patients who had died within 6 months of follow-up, and adjusted for indicators of nutritional status (e.g., BMI, total cholesterol, hemoglobin, and serum albumin) and comorbidities (hypertension, diabetes, CV disease, CCI) to make the results more reliable. This association was more pronounced in the older adults with normal kidney function or proteinuria, suggesting that low SUA acts as an independent biological indicator of CV disease in the absence of kidney decline. While CKD itself is an independent risk factor for CV disease [19], SUA is more likely to be a biological indicator of kidney function when combined with CKD. In addition, our study further provide definitive evidence for the association between asymptomatic hyperuricemia (without gout) and mortality in older people, and that was not attenuated after adjusting for kidney function and other CV disease risk factors.

Our study is suggestive of the two important but opposing properties of SUA. The putative toxic mechanism of hyperuricemia is now largely accepted to include stimulation of inflammation [20], induction of endothelial cell dysfunction [21], stimulation of vascular smooth muscle proliferation [22], and increasing oxidative stress [23], which may contribute to a substantially higher CV disease risk in these patients. A previous study reported that uric acid crystals are present in the aorta and coronary vessels of subjects, and that many uric acid deposits correspond to sites of vascular calcification and plaque formation [24], which may contribute to a substantially higher CV disease risk in these patients. However, uric acid also exerts antioxidant actions in human plasma and plays a protective role for vascular cells, including stabilization of endothelial nitric oxide synthase activity [25]. SUA may protect against various neurodegenerative diseases such as Parkinson’s disease, Alzheimer’s disease, and amyotrophic lateral sclerosis [26, 27]. The activity of enzymatic antioxidants decreases with age [28], hence, older adults are more likely to rely on non-enzymatic compounds, primarily uric acid (UA), for antioxidant action. Our results suggest that dichotomous classification of the SUA concentration is not appropriate because the mortality risk posed by an extremely low SUA concentration may be masked by this classification in future studies.

This is a prospective study design, a relatively large sample size, and a nationally representative sample of older people, which facilitates generalization. Moreover, by using 1 mg/dL increments to stratify patients according to SUA concentration rather than using quantiles, our findings make it easier for patients and physicians to standardize individual values. Finally, we adjusted for multiple potential confounders based on the understanding of elderly population, that improved the validity and robustness of our conclusions.

Several potential limitations of this study should be acknowledged. First, because it was an observational study, residual confounding factors are possible. Second, this study was conducted based on a database of only US patients; therefore, caution should be taken when generalizing the finding to other ethnicities. Third, SUA concentrations were measured only once, which may underestimate the true association of interest [29]. Finally, following the recognition that SUA concentration plays an important role in older people, controlling uric acid to an appropriate range is expected to reduce the burden of these patients; however, our study design cannot shed further light on this issue.

## Conclusions

Our analyses suggested that both low and high SUA concentrations are associated with an increased risk in all-cause and CV mortality among older people. Extremely low SUA concentrations are especially undesirable, especially in those with normal kidney function.

## Data Availability

The datasets generated and analyzed in the current study are available at NHANES website: https://www.cdc.gov/nchs/nhanes/index.htm.
